# Exposure to interparental violence and justification of intimate partner violence among women in Papua New Guinea

**DOI:** 10.1186/s12905-023-02248-9

**Published:** 2023-03-23

**Authors:** Richard Gyan Aboagye, Bernard Yeboah-Asiamah Asare, Collins Adu, Abdul Cadri, Abdul-Aziz Seidu, Bright Opoku Ahinkorah, Sanni Yaya

**Affiliations:** 1 Department of Family and Community Health, Fred N. Binka School of Public Health, University of Health and Allied Sciences, Hohoe, Ghana; 2 Curtin School of Population Health, Curtin University, Perth, Australia; 3 Institute of Applied Health Sciences, University of Aberdeen, Aberdeen, Scotland UK; 4 College of Public Health, Medical and Veterinary Sciences, James Cook University, Townsville, QLD 4811 Australia; 5 Center for Social Research in Health, University of New South Wales Sydney, Sydney, NSW 2052 Australia; 6 Department of Social and Behavioural Science, School of Public Health, University of Ghana, Legon- Accra, Ghana; 7 Department of Family Medicine, Faculty of Medicine, McGill University, Montreal, QC Canada; 8 Centre For Gender and Advocacy, Takoradi Technical University, P.O.Box 256, Takoradi, Ghana; 9 School of Public Health, Faculty of Health, University of Technology Sydney, Sydney, Australia; 10 School of International Development and Global Studies, University of Ottawa, Ottawa, Canada; 11 The George Institute for Global Health, Imperial College London, London, UK

**Keywords:** Interparental violence, Intimate partner violence, Papua New Guinea, Global Health

## Abstract

**Background:**

Previous studies have demonstrated that exposure to interparental violence is associated with intimate partner violence justification in a variety of contexts. In this study, we examined the association between exposure to interparental violence and justification of intimate partner violence among women in Papua New Guinea.

**Methods:**

We used data from the 2016–18 Papua New Guinea Demographic and Health Survey. We included 2839 women of reproductive age (15–49 years) in a sexual union (married and cohabiting) in the study. We used a multivariable binary multilevel regression analysis to examine the association between interparental violence and justification of intimate partner violence. We presented the results of the regression analysis using crude odds ratio (cOR) and adjusted odds ratios (aORs), with their 95% confidence intervals (CIs).

**Results:**

Women exposed to interparental violence were 1.26 (95%CI = 1.05, 1.53) times more likely to justify intimate partner violence than those who were not exposed. Women who resided in the Highlands (aOR = 2.50, 95%CI = 1.78, 3.51), Momase (aOR = 1.96, 95%CI = 1.40, 2.75), and Islands (aOR = 1.42, 95%CI = 1.01, 1.99) were more likely to justify intimate partner violence compared to those in the Southern region. Women who were exposed to one (aOR = 1.38, 95%CI = 1.06, 1.82) mass media were more likely to justify intimate partner violence compared to those who had no exposure to mass media. On the other hand, women aged 25–34 years (aOR = 0.66, 95%CI = 0.48, 0.91) and 35–49 years (aOR = 0.66, 95%CI = 0.44, 0.97) were less likely to justify intimate partner violence compared to those aged 15–24 years.

**Conclusions:**

Our study has shown that exposure to interparental violence is a predictor of intimate partner violence justification. This study suggests the need for conscious and continuous efforts to identify and assist women who have been exposed to interparental violence to help prevent its transition to later life. Policies and interventions should be developed and implemented to curtail children’s exposure to domestic violence in their households. Also, laws and policies need to condemn any violence and demystify community justification and acceptance of intimate partner violence, taking into consideration the significant sociodemographic characteristics of the women highlighted in the study.

## Introduction

Intimate partner violence (IPV), encompasses a variety of sexual, emotional, and physical coercive behaviours committed in the context of an intimate relationship [1]. It has emerged as a major public health concern worldwide. IPV has been widely documented against both men and women around the world [2], with both sexes being either perpetrators or victims. Nevertheless, studies have reported that men are more likely to be the perpetrators of severe kinds of IPV [3–5]. Sabri et al. [6] posits that IPV can increase the risk of sexually transmitted infections and Human Immunodeficiency Virus (HIV) by limiting a victim's ability to negotiate safer sex because of fear of further violence. Even though it is also a violation of human rights [7], it is also a social issue that has a negative effect on economic empowerment, especially for women who are victims.

A population-based IPV survey conducted in India in 2011 found that about 8,618 women died as a result of intimate partner abuse [8]. IPV continues to be a significant threat to women's lives around the world, as well as an obstacle to empowering women as part of Sustainable Development Goal (SDG) 5 [9]. Historically, women have been the primary victims of IPV in all countries [4, 5, 10, 11]. Almost one-third (27%) of women aged 15 to 49 years worldwide have experienced lifetime intimate relationship abuse [7]. While it is a huge problem around the world, data shows that it's becoming more common in many developing Asian and African countries [7].

Previous studies have demonstrated that exposure to interparental violence is a substantial predictor of IPV justification in a variety of circumstances [1, 12, 13]. There is evidence that women's socioeconomic status (wealth index and education level) influences how women justify IPV [14]. In sub-Saharan Africa (SSA), Aboagye et al. [12] found that women who had been subjected to interparental violence were more likely to justify IPV than those who had not been subjected. In Ghana, Adu [14] found that women with higher education levels and those who were wealthier were less likely to defend intimate partner abuse.

High levels of IPV justification have been seen in both male and female partners in various circumstances [12, 15], with women being more prone to justify IPV [12, 15]. In Papua New Guinea (PNG), IPV affects more than 80% of women, a rate that is thought to be one of the highest in the world and can occasionally result in serious bodily harm [16–18]. About 41% of men reported raping their wives within the preceding year, according to a study on gender-based violence in PNG [16]. In PNG, the prevalence of spousal rape rose to about 87.3% [19]. According to Adu et al. [11], the majority of IPV instances in PNG are rarely recorded, and as a result, there is a pervasive and stifling silence regarding how IPV affects women. The current climate of emotional, physical, and sexual violence in PNG violate women's basic human rights, causes untold pain and misery, impedes women's participation in the country's development, reinforces other forms of violence in society, and psychologically harms children who witness such violence [20].

Understanding the magnitude and exposure of interparental violence linked with IPV justification is a critical prerequisite for designing successful interventions to address IPV against women in society. In view of this, we examined the association between exposure to interparental violence and IPV justification among women in PNG using a nationally representative dataset. Findings from the study will help improve interventions aimed at reducing IPV in PNG.

## Methods

### Data source and study design

We used data from the 2016–18 PNG Demographic and Health Survey (DHS). The data were extracted from the women’s file (individual recode file). DHS is a nationally representative survey conducted in over 85 low-and-middle-income countries globally since its inception [21]. Specifically, the 2016–18 PNG DHS was conducted to provide up-to-date estimates of demographic and health indicators, including domestic violence [21]. A cross-sectional design was used in the DHS. The data were collected from the respondents using standardized structured interviewer-administered questionnaires. Respondents for the DHS were sampled using a stratified two-stage cluster sampling technique. Clusters were chosen in the first step using a probability proportional to size sampling approach. In the second stage, a systematic sampling technique was used to select a predetermined number of households (usually 28–30). Detailed sampling technique has been highlighted in the literature [22]. We included 2839 women of reproductive age (15–49 years) in a sexual union (married and cohabiting) who had complete data on all the variables of interest in the study. The dataset used is freely available to download at https://dhsprogram.com/data/dataset_admin/index.cfm. We based on the Strengthening the Reporting of Observational Studies in Epidemiology (STROBE) guidelines in drafting this paper [23].

### Variables

Justification of IPV was the outcome variable in the study. We estimated IPV justification using the responses to five questions regarding their husband’s/partner’s justification of wife-beatings. The five instances under which wives were beaten include (i) burning food, (ii) arguing with him, (iii) going out without telling him, (iv) neglecting the children, and (v) refusing to have sexual intercourse with him. The response options as found in the DHS were “no”, “yes”, and “don’t know”. We dropped all those who responded “don’t know”. Afterward, women whose response option was “yes” in any of the five items were said to have justified IPV whilst those with “no” responses in all the items were categorized as not justifying IPV [10–12, 24–26].

We considered exposure to interparental violence as the key explanatory variable in our study. With this variable, the women were asked “*As far as you know, did your father ever beat your mother?”.* The response options were “no”, “yes”, and “don't know”. We dropped those who responded “don't know”. The response option “yes” was recoded as exposed to interparental violence. The response option "no" was recoded as not exposed to interparental violence. Studies using the DHS dataset have utilized similar coding and categorization [1, 12, 27].

Based on a review of the literature [10–12, 24–26], we included twelve variables as covariates in the study. Also, the covariates were selected based on their availability in the PNG DHS. We grouped the covariates into individual-level and household/community-level variables, respectively. The individual-level covariates consisted of the age of the women, educational level, current working status, marital status, parity, and exposure to mass media. We utilized the existing coding for the current working status (no and yes) as found in the DHS. We recoded age of women into “15–24”, “25–34”, and “35–49”. The women’s partners age was coded as “15–24”, “25–34”, “35–44”, and “45 + ”. Level of education of the women and their partners was recoded as “no education”, “primary”, and “secondary or higher”. Parity was coded as “zero birth”, “one birth”, “two births”, “three births”, and “four or more birth”. Marital status was coded into “married” and “cohabiting”. Exposure to mass media was created as an index variable from frequency of listening to radio, frequency of watching television, and frequency of reading newspaper or magazine. The responses in each of the variable were “not at all”, “less than once a week”, and “at least once a week”. Women whose response options were “not at all” were recoded as not exposed (no) whilst the remaining response options were recoded as exposed (yes) in each of the three variables. Based on the recoded responses, a new variable called the mass media exposure was created with the categories being “none [not exposed to any of the three variables])”, “one [exposed to only one of the three variables]”, and “two or more [exposed to at least two of the three variables]”. Wealth index, place of residence, region, community socioeconomic status, and community literacy level were the household/community level covariates in the study. We maintained the existing coding for wealth index (poorest, poorer, middle, richer, and richest), sex of household head (male and female), place of residence (urban and rural), and region (southern, Highlands, Momase, and Islands) as found in the DHS. Community literacy level and community socioeconomic status were categorized into “low”, “medium”, and “high”.

### Statistical analyses

**Fig. 1:**
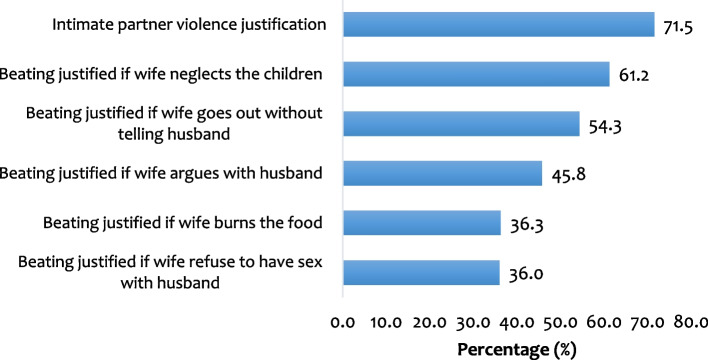
Prevalence of intimate partner violence justification among the women in Papua New Guinea

Stata software version 16.0 (Stata Corporation, College Station, TX, USA) was used to perform the statistical analysis. The proportion of IPV justification among the women was expressed as a percentage (Fig. 1). Using a cross-tabulation, we looked at the distribution of IPV justification across exposure to interparental violence and the covariates (Table 1). To examine the association between interparental violence exposure and IPV justification, we utilized a multivariable binary multilevel regression analysis. We checked for evidence of collinearity among the study variables using the variance inflation factor (VIF). The results showed no evidence of collinearity among the variables (minimum VIF = 1.03, maximum VIF = 3.96, and mean VIF = 2.63). We used five models to examine the association between interparental violence exposure and IPV justification, controlling for the covariates. Model O (empty model) was created to determine how the clustering of the primary sample units affected the IPV justification. In Model I, we included only the key explanatory variable (exposure to interparental violence) and the IPV justification. We placed the key explanatory variable, the individual-level covariates and IPV justification in Model II. Model III contained the key explanatory variable, household/community level covariates, and IPV justification. Finally, in Model IV, we included all the explanatory variables (key explanatory, individual-level, and community-level variables, respectively) and IPV justification. We presented the results of the regression analysis using crude odds ratio (cOR) and adjusted odds ratios (aOR), with their 95% confidence intervals (CIs). Statistical significance was set at p < 0.05. To evaluate model fitness and comparability, the Akaike Information Criterion (AIC) was utilized. The model with the least AIC value was selected as the best-fitted model for the study. All of the analyses were weighted to account for over-and under-sampling, non-response, and to increase the generalizability of the findings. The Stata command "svyset" was used in all analyses.

**Table 1:** Distribution of intimate partner violence justification across the explanatory variables

Variable	Weighted		IPV Justification	
	**Frequency**	**Percentage**	**Yes**	***P*-value**
**Exposed to interparental violence**				0.014
No	1474	51.9	68.9 [65.4, 72.1]	
Yes	1365	48.1	74.4 [71.1, 77.4]	
**Women’s age (years)**	(Mean = 32.5;SD = 7.90)			
**Women’s age**				0.039
15–24	581	20.5	78.7 [7.19, 84.2]	
25–34	1148	40.4	71.3 [67.2, 75.0]	
35–49	1110	39.1	68.0 [63.3, 72.4]	
**Women’s educational level**				0.523
No education	760	26.8	70.8 [65.2, 75.8]	
Primary	1431	50.4	72.7 [69.4, 75.9]	
Secondary or higher	648	22.8	69.7 [65.5, 73.5]	
**Marital status**				0.112
Married	2311	81.4	70.7 [67.8, 73.4]	
Cohabiting	528	18.6	75.3 [70.2, 79.8]	
**Current working status**				0.739
No	1912	67.3	71.8 [68.8, 74.7]	
Yes	927	32.7	70.9 [66.2, 75.2]	
**Parity**				0.052
Zero birth	241	8.5	79.8 [72.8, 85.4]	
1 birth	500	17.6	75.4 [67.7, 81.7]	
2 births	476	16.8	73.7 [67.4, 79.2]	
3 births	471	16.6	72.0 [66.8, 76.7]	
4 or more births	1151	40.5	67.0 [62.2, 71.5]	
**Exposure to mass media**				0.067
None	1519	53.5	69.2 [65.8, 72.4]	
One	545	19.2	75.5 [70.1, 80.2]	
Two or more	775	27.3	73.3 [69.2, 77.0]	
**Partner's educational level**				0.296
No education	561	19.8	67.8 [61.2, 73.8]	
Primary	1309	46.1	72.2 [68.6, 75.4]	
Secondary or higher	969	34.1	72.8 [69.3, 76.0]	
**Partner's age**				0.005
15–24	180	6.3	86.1 [78.7, 91.2]	
25–34	1042	36.7	70.5 [65.3, 75.2]	
35–44	994	35.0	73.8 [69.5, 77.8]	
45 +	623	21.9	65.3 [60.2, 70.0]	
**Wealth index**				0.908
Poorest	575	20.3	70.4 [64.6, 75.7]	
Poorer	558	19.7	71.3 [65.3, 76.6]	
Middle	617	21.7	70.7 [66.0, 75.1]	
Richer	560	19.7	71.7 [66.5, 76.5]	
Richest	529	18.6	73.6 [69.2, 77.6]	
**Place of residence**				0.931
Urban	287	10.1	71.7 [66.6, 76.4]	
Rural	2552	89.9	71.5 [68.8, 74.1]	
**Region**				< 0.001
Southern Region	509	17.9	61.2 [56.6, 65.6]	
Highlands Region	1021	36.0	75.0 [70.3, 79.2]	
Momase Region	861	30.3	74.9 [70.6, 78.8]	
Islands Region	448	15.8	68.8 [62.5, 74.5]	
**Community literacy level**				0.712
Low	1342	47.3	71.2 [67.0, 75.0]	
Medium	784	27.6	73.0 [68.5, 77.2]	
High	713	25.1	70.5 [66.1, 74.5]	
**Community socioeconomic status**				0.575
Low	1718	60.5	70.6 [67.3, 73.6]	
Medium	253	8.9	75.1 [64.6, 83.3]	
High	868	30.6	72.4 [68.2, 76.2]	

*SD* Standard deviation, *P*-values were generated from the chi-square test

### Ethical consideration

We did not seek ethical approval for this study since the dataset is available in the public domain. However, we sought permission from the MEASURE DHS before using the dataset and it was granted. We adhered to the ethical guidelines regarding the use of secondary dataset for publication. The detailed information concerning the ethical guidelines can be accessed at http://goo.gl/ny8T6X.

## Results

### Background characteristics of the respondents

Table 1 presents the background characteristics of the respondents. The mean age of the respondents was 32.5 (SD = 7.90) years. Most of the women were aged 25–34 (40.4%). Majority of the women were married (81.4%), and had 4 or more births (40.5%). Most of the women resided in rural areas (89.9%) and in the Highlands Region (36.0%). Most of the women had attained primary school education (46.1%), currently not working (67.3%), and not exposed to mass media (53.5%). Most of the women resided in communities with low literacy level (47.3%) and socioeconomic status (60.5%) and were in the poorest wealth index (20.3%) (Table 1).

### Prevalence of intimate partner violence justification among women in Papua New Guinea

Figure 1 shows the prevalence of IPV justification among women in sexual union in PNG. Overall, 71.5% (95% CI = 69.0, 73.9) of the women justified IPV. More than half of the women justified the partner’s beating if the wife neglects the children (61.2%) and goes out without informing or the permission of the husband (54.3%). Approximately 46% of the women also indicated beating of a wife is justified if the wife argues with the husband and about a third of women cited burning of food (36.3%) and refusal to have sex with the husband (36.0%) as justification of IPV.

### Distribution of intimate partner violence justification across the explanatory variables

Tables 1 present the distribution of IPV justification across exposure to interparental violence and the covariates explanatory variables. Less than half of the women (48.1%) reported having been exposed to interparental violence. Most of the women who were exposed to interparental violence (74.4%) indicated justification of IPV. The Chi-square test showed a significantly high proportion of women exposed to interparental violence justified IPV than among women who had not been exposed to interparental violence (74.4% vs 68.9%, p = 0.014). Except for exposure to interparental violence, women’s age, partners age, and region, all the remaining variables had no statistically significant relationship with the justification of IPV (Table 1).

### Association between exposure to interparental violence and intimate partner violence justification

Table 2 presents the results of the association between exposure to interparental violence and IPV justification. In Model I, without adjusting for covariates, women who were exposed to interparental violence were more likely to justify IPV (cOR = 1.29; (95%CI = 1.07, 1.55) compared to those who were not exposed. After adjusting for all the covariates, women exposed to interparental violence had high odds of justifying IPV [aOR = 1.26; (95%CI = 1.05, 1.53)] compared to those who were not exposed.

**Table 2:** Association between exposure to interparental violence and intimate partner violence justification in Papua New Guinea

Variable	Model O	Model I cOR [95% CI]	Model II aOR [95% CI]	Model III aOR [95% CI]	Model IV aOR [95% CI]
**Fixed effect**					
**Exposed to inter-parental violence**					
No		1.00	1.00	1.00	1.00
Yes		1.29^**^ [1.07, 1.55]	1.29^**^ [1.06, 1.55]	1.27^*^ [1.06, 1.54]	1.26^*^ [1.05, 1.53]
**Women’s age**					
15–24			1.00		1.00
25–34			0.65^*^ [0.47, 0.90]		0.66^*^ [0.48, 0.91]
35–49			0.65^*^ [0.44, 0.96]		0.66^*^ [0.44, 0.97]
**Women’s educational level**					
No education			1.00		1.00
Primary			0.93 [0.71, 1.21]		1.01 [0.76, 1.33]
Secondary or higher			0.73 [0.52, 1.02]		0.79 [0.55, 1.13]
**Marital status**					
Married			1.00		1.00
Cohabiting			1.11 [0.86, 1.44]		1.05 [0.81, 1.37]
**Current working status**					
No			1.00		1.00
Yes			0.95 [0.77, 1.17]		0.99 [0.80, 1.22]
**Parity**					
Zero birth			1.00		1.00
1 birth			0.83 [0.55, 1.25]		0.85 [0.57, 1.28]
2 births			0.95 [0.64, 1.43]		1.00 [0.67, 1.49]
3 births			0.94 [0.63, 1.41]		0.98 [0.66, 1.47]
4 or more births			0.97 [0.66, 1.43]		1.04 [0.71, 1.53]
**Exposure to mass media**					
None			1.00		1.00
One			1.36^*^ [1.04, 1.78]		1.38^*^ [1.06, 1.82]
Two or more			1.28 [0.98, 1.68]		1.26 [0.95, 1.67]
**Partner's educational level**					
No education			1.00		1.00
Primary			1.16 [0.87, 1.55]		1.30 [0.96, 1.74]
Secondary or higher			1.15 [0.83, 1.58]		1.22 [0.87, 1.70]
**Partner's age**					
15–24			1.00		1.00
25–34			0.79 [0.49, 1.29]		0.79 [0.49, 1.28]
35–44			0.74 [0.44, 1.25]		0.72 [0.43, 1.21]
45 +			0.58 [0.33, 1.01]		0.55^*^ [0.32, 0.96]
**Wealth index**					
Poorest				1.00	1.00
Poorer				1.00 [0.71, 1.39]	0.99 [0.71, 1.38]
Middle				0.88 [0.63, 1.22]	0.84 [0.59, 1.18]
Richer				0.97 [0.67, 1.40]	0.93 [0.63, 1.36]
Richest				0.98 [0.63, 1.52]	1.00 [0.62, 1.61]
**Place of residence**					
Urban				1.00	1.00
Rural				0.80 [0.55, 1.18]	0.82 [0.56, 1.21]
**Region**					
Southern Region				1.00	1.00
Highlands Region				2.28^***^ [1.64, 3.16]	2.50^***^ [1.78, 3.51]
Momase Region				1.88^***^ [1.35, 2.62]	1.96^***^ [1.40, 2.75]
Islands Region				1.34 [0.96, 1.88]	1.42^*^ [1.01, 1.99]
**Community literacy level**					
Low				1.00	1.00
Medium				1.18 [0.86, 1.61]	1.16 [0.84, 1.61]
High				1.05 [0.75, 1.48]	1.06 [0.74, 1.53]
**Community socioeconomic status**					
Low				1.00	1.00
Medium				1.41 [0.87, 2.27]	1.40 [0.87, 2.26]
High				1.03 [0.76, 1.41]	1.00 [0.73, 1.37]
**Random effect result**					
PSU variance (95% CI)	0.994 [0.711, 1.390]	0.957 [0.680, 1.346]	0.972 [0.6885, 1.372]	0.847 [0.589, 1.218]	0.845 [0.584, 1.223]
ICC	0.232	0.225	0.228	0.205	0.204
LR Test	94.03 (< 0.001)	88.71 (< 0.001)	86.54 (< 0.001)	72.09 (< 0.001)	68.80 (< 0.001)
Wald chi-square	Reference	7.03 (0.008)	41.03 (0.001)	39.38 (< 0.001)	75.27 (< 0.001)
**Model fitness**					
Log-likelihood	-1716.1022	-1712.5932	-1694.7094	-1696.364	-1676.5866
AIC	3436.204	3431.186	3429.419	3422.728	3417.173
N	2839	2839	2839	2839	2839
Number of clusters	721	721	721	721	721

*aOR* adjusted odds ratios, *CI* Confidence interval, *cOR* Crude odds ratio

^*^ *p* < 0.05

^**^ *p* < 0.01

^***^ *p* < 0.001; 1.00 = Reference category, *PSU* Primary sampling unit, *ICC* Intra-class correlation, *LR* Test = Likelihood ratio test, *AIC* Akaike’s information criterion

The results on other covariates showed women who resided in the Highlands (aOR = 2.50, 95%CI = 1.78, 3.51), Momase (aOR = 1.96, 95%CI = 1.40, 2.75), and Islands (aOR = 1.42, 95%CI = 1.01, 1.99) were more likely to justify IPV compared to those in the Southern region. Women who were exposed to one (aOR = 1.38, 95%CI = 1.06, 1.82) mass media were more likely to justify IPV compared to those who had no exposure to mass media. On the other hand, women aged 25–34 (aOR = 0.66, 95%CI = 0.48, 0.91) and 35–49 (aOR = 0.66, 95%CI = 0.44, 0.97) were less likely to justify IPV compared to those aged 15–24 years.

## Discussion

This study examined the association between exposure to interparental violence and IPV justification among women in PNG. We found that 71.5% of the women justify IPV, and being exposed to interparental violence increased the likelihood of women justifying IPV. The rate of IPV justification among women found in this study is higher than reported among married women in Bangladash (32.4%-46.5%) [28, 29], and Turkey (41%) [30] but comparable to the rates seen in some countries in such as Ethiopia (74%) [31] and Mali (76.6%) [32]. Consistent with previous studies [28, 31–33], “beating justified if wife neglects children”, “beating justified if wife goes out without telling husband”, and “beating justified if wife argues with husband” were mostly indicated as reasons for justifying IPV. Justification of IPV among women is indicated to be high among women around the world, particularly in communities where IPV is common [28], which is the case in PNG [16].

We found that women who have been exposed to interparental violence were more likely to justify IPV in PNG. This is consistent with findings from several low-and middle-income countries [12, 28, 34]. Children are reported to learn by observing their parents and emulating their behaviors from childhood to adulthood [28]; hence, women’s justification of IPV could be nurtured from witnessing the abuse of their mother and the mother’s attitude of accepting and normalizing such violence over time [1]. Our finding suggests that in order to reduce exposure to violence for future generations, interventions may focus on preventing IPV. Interventions could include educational campaigns that increase awareness about IPV and its related consequences and encourage married partners in identifying and avoiding such behaviors.

Women who were older were less likely to justify IPV. Similar findings have been reported in other low-and middle-income countries [34, 35]. Women may acquire more education as they get old increasing their awareness and insights on IPV and may change their attitudes toward IPV as they become older. Older women may also have accumulated/cultivated self-esteem, self-reliance, and self-confidence in their relationships over the years [32].

Women who were exposed to the mass media were found to justify IPV. Consistent findings have been reported in Mali [32], but in contrast to the finding reported in Ghana, where IPV justification was less likely among women exposed to mass media [36]. The mass media have become platforms for social discourse and could be that in societies where IPV is common and as such normalized/accepted [28], such behaviours may be propagated through these media. However, the significance of the mass media in promoting equality and social inclusion [37] could be used in rolling out educational campaigns to increase awareness about IPV and its associated negative consequences and demystify community justification or acceptance of such behaviours.

IPV is indicated to be more common in less developed regions [1, 38]. Consistent with our study, women who resided in Highlands, Momase, and Islands regions were more likely to justify IPV compared to those from the Southern region, which is the National capital region and may be more developed than the other regions. As such, women from the Southern region of PNG may have access to high education and employment, which could help increase their awareness about the negative effects of IPV and empower them.

### Strength and limitations

This study was drawn on data from a nationally representative and large sample thereby enhancing the rigour of the study and the generalizability of the findings. Some limitations to the study are however noted. The use of a cross-sectional study design limits the drawing of any causal inferences from the findings. In addition, using self-reported data suggests there could be an issue with recall and social desirability bias which may result in the under-or over-reporting of the study variables, particularly justification of IPV.

## Conclusions

This study has demonstrated that women’s exposure to interparental violence is a significant predictor of IPV justification in later life. Our study has also shown that women of older ages were less likely to justify IPV; whereas those who resided in Highlands, Momase, and Islands regions, and those who were frequently exposed to the mass media were more likely to justify IPV. This study suggests the need for conscious and continuous efforts to identify and assist women who have been exposed to interparental violence to help prevent its transition to later life. Policies and interventions should be developed and implemented to curtail children’s exposure to domestic violence in their households. Also, laws and policies need to condemn any violence and demystify community justification and acceptance of intimate partner violence, taking into consideration the significant sociodemographic characteristics of the women highlighted in the study.

## Data Availability

The dataset used is available at https://dhsprogram.com/data/dataset/Papua-New-Guinea_Standard-DHS_2017.cfm?flag=1

## Abbreviations

AIC: Akaike information criterion
aOR: Adjusted odds ratio
BIC: Bayesian information criterion
CI: Confidence interval
DHS: Demographic and health survey
HIV: Human immunodeficiency virus
ICC: Inter-cluster correlation
ICF: Inner city fund
IPV: Intimate partner violence
PNG: Papua new Guinea
STROBE: Strengthening reporting of observational studies in epidemiology
VIF: Variance inflation factor
